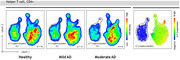# Stage‐Dependent Reduction of ILT‐2 Negative Helper T Cells in Alzheimer's Disease: UMAP Insights from PBMCs

**DOI:** 10.1002/alz70855_098133

**Published:** 2025-12-23

**Authors:** Cheng‐I Chu, Ching‐Tse Wu, Hui‐Yu Yang, Chien‐Chung Chang, Chuang‐Rung Chang

**Affiliations:** ^1^ National Taiwan University Hospital Hsinchu Branch, Hsinchu, Taiwan; ^2^ National Tsing Hua University, Hsinchu, Taiwan

## Abstract

**Background:**

The immune system, particularly through chronic inflammation and immune dysfunction, plays a crucial role in Alzheimer's disease(AD) development. Impairment of helper T cells may accelerate AD progression. ILT‐2(Immunoglobulin‐like Transcript 2) is an inhibitory receptor on T cells that regulates immune activity. Our high‐dimensional screening unexpectedly revealed significant changes in ILT‐2 negative helper T cell populations in AD patients. Using UMAP (Uniform Manifold Approximation and Projection) to visualize the data, we examined these changes in peripheral blood mononuclear cells (PBMCs). This study explores the relationship between ILT‐2 expression and AD severity, providing insights into immune mechanisms in AD progression.

**Method:**

We enrolled 17 AD patients (11 with mild AD, CDR=1; 6 with moderate AD, CDR=2) and 16 healthy controls. Peripheral blood was collected, and white blood cells were labeled with fluorescent antibodies for flow cytometry analysis. UMAP was used for high‐dimensional reduction to examine T‐cell populations and their functional characteristics. Data from all subjects were aggregated and analyzed to compare feature distribution patterns across AD stages and healthy controls, identifying molecular features for further subpopulation analysis.

**Result:**

High‐dimensional analysis of helper T cell (CD4+) populations, based on multiple surface protein expressions, showed differences in subpopulation distributions between mild AD, moderate AD, and healthy elderly individuals. These differences are associated with ILT‐2 expression. The ratio of ILT‐2 negative helper T cell population was reduced in both mild and moderate AD PBMCs compared to healthy controls. Moderate AD patients exhibited an even smaller ILT‐2 negative helper T cell population than those with mild AD.

**Conclusion:**

ILT‐2, traditionally an immune checkpoint receptor on T cells, may exhibit a cytotoxic phenotype in CD4+ILT‐2+ T cells, playing a role in cancer cell cytotoxicity. In AD patients, we observed a decreased proportion of ILT‐2 negative helper T cells and an increase in the CD4+ILT‐2+ population, indicating enhanced cytotoxic function within the helper T cell compartment. Our findings highlight an immune imbalance in AD patients, with significant alterations in T cell subpopulations. These changes could serve as potential biomarkers for AD progression, and ILT‐2 emerges as a promising target for future therapeutic interventions in AD.